# B chromosomes reveal a female meiotic drive suppression system in *Drosophila melanogaster*

**DOI:** 10.1016/j.cub.2023.04.028

**Published:** 2023-05-04

**Authors:** Stacey L. Hanlon, R. Scott Hawley

**Affiliations:** 1 Genetics and Genomics, Department of Molecular and Cell Biology, University of Connecticut, Storrs, CT 06269, USA; 2 Institute for Systems Genomics, University of Connecticut, Storrs, CT 06269, USA; 3 Stowers Institute for Medical Research, Kansas City, MO 64110, USA; 4 Department of Molecular and Integrative Physiology, University of Kansas Medical Center, Kansas City, KS 66160, USA; 5 Twitter: @DNAdevotee; 6 Lead contact

## Abstract

Selfish genetic elements use a myriad of mechanisms to drive their inheritance and ensure their survival into the next generation, often at a fitness cost to its host.^1,2^ Although the catalog of selfish genetic elements is rapidly growing, our understanding of host drive suppression systems that counteract self-seeking behavior is lacking. Here, we demonstrate that the biased transmission of the non-essential, non-driving B chromosomes in *Drosophila melanogaster* can be achieved in a specific genetic background. Combining a null mutant of *matrimony*, a gene that encodes a female-specific meiotic regulator of Polo kinase,^3,4^ with the *TM3* balancer chromosome creates a driving genotype that is permissive for the biased transmission of the B chromosomes. This drive is female-specific, and both genetic components are necessary, but not individually sufficient, for permitting a strong drive of the B chromosomes. Examination of metaphase I oocytes reveals that B chromosome localization within the DNA mass is mostly abnormal when drive is the strongest, indicating a failure of the mechanism(s) responsible for the proper distribution of B chromosomes. We propose that some proteins important for proper chromosome segregation during meiosis, like Matrimony, may have an essential role as part of a meiotic drive suppression system that modulates chromosome segregation to prevent genetic elements from exploiting the inherent asymmetry of female meiosis.

## In brief

B chromosomes are genetic renegades that can rapidly spread through a population when left unchecked. Hanlon and Hawley show that the B chromosomes in *D. melanogaster* are subject to female meiotic drive in a specific mutant, leading to the identification of a drive suppression system that may protect against invasion by selfish genetic elements.

## Graphical abstract

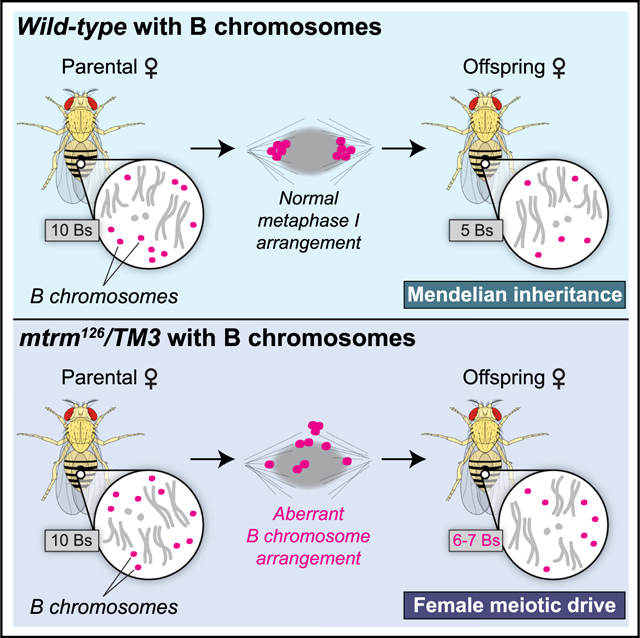

## RESULTS AND DISCUSSION

Mendel’s first law of segregation dictates that gametes will receive one of the two alleles at random for a given genetic locus and that the ratio of each allele observed in the resulting progeny will be close to one-to-one. This tenet only holds if genetic inheritance remains unbiased, an assumption that is rapidly accumulating exceptions as more genetic elements that act selfishly are discovered.^2,5–7^ Supernumerary B chromosomes are model selfish genetic elements since they are not essential for the normal growth and development of an organism yet can be stably maintained in a population.^8,9^ To accomplish this feat, many B chromosomes have been shown to bias their transmission to progeny to ensure their propagation and maintenance over many generations, a phenomenon referred to as drive.^10–18^

Recently, B chromosomes were found to have arisen in a single laboratory stock of *Drosophila melanogaster* (*D. melanogaster*).^19^ These B chromosomes are gene poor, are unable to generate their own drive, and impose a fitness cost because they promote high levels of chromosome 4 missegregation during meiosis.^19,20^ In just under 20 years, however, these B chromosomes proliferated through their original stock at a rapid pace to reach an equilibrium of 10–12 B chromosomes in addition to the four pairs of essential chromosomes. This original stock carries two genetically engineered aberrations that may contribute to the biased inheritance of the B chromosomes: a null allele of *matrimony* (*mtrm*^126^), which binds to Polo kinase to govern the distributive (achiasmate) system that acts as a backup system for segregating chromosomes that did not form crossovers,^3,4,21–24^ and *TM3* , a multiply inverted third chromosome balancer that has the potential to disrupt the normal segregation of other chromosomes during female meiosis.^25^

### B chromosomes are preferentially transmitted by females in the original stock

**Figure 1.**
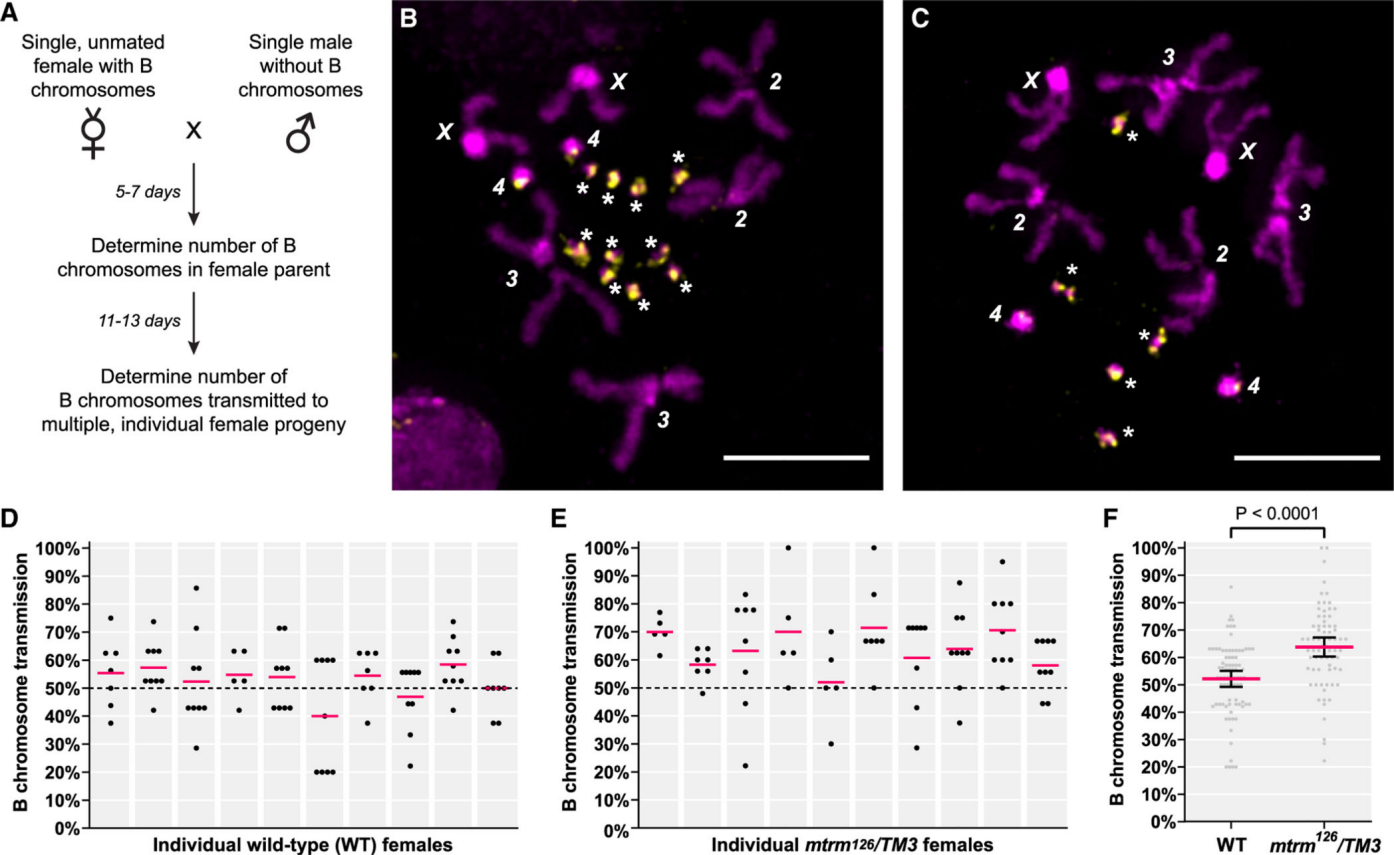
Cytological assessment of the adult female germline reveals that B chromosomes are subject to biased transmission in their original stock (A) Workflow for evaluating B chromosome transmission in a single female. (B and C) Representative image of a metaphase from an ovary mitotic preparation from a parental female with ten B chromosomes (B) and one of her daughters with five B chromosomes (C). In this example, the B chromosome transmission was 50%. The B chromosomes (labeled with *) are readily identified using a FISH probe that recognizes the *AAGAT* satellite sequence (yellow), which is enriched on the B chromosomes. DNA is in magenta (DAPI). Scale bars, 5 μm. (D) Transmission frequencies of B chromosomes between wild-type (WT) parental females and their progeny, plotted by individual females. The red line indicates the mean transmission for that individual parental female. The dotted line is set at 50%, which is the expected transmission if B chromosome segregation was random. (E) Same as in (D) but parental females are from the original *mtrm*^*126*^*/TM3* B chromosome stock. Within each genotype, there was no significant difference between the means of the parental females (Welch’s ANOVA produced a non-significant p value of 0.3789 for WT and 0.0744 for *mtrm*^*126*^*/TM3* females). (F) Cumulative transmission frequency of B chromosomes in females with a WT (n = 81) or original *mtrm*^*126*^*/TM3* stock (n = 72) genotype. The red line indicates the mean, and the error bars represent the 95% confidence interval of the mean. The p value is indicated in the figure (unpaired t test with Welch’s correction). See also Figure S1 and Data S1A–S1E.

We first focused on whether the *mtrm*^*126*^*/TM3* stock (herein referred to as the “original stock”) can support the drive of the B chromosomes. To assess B chromosome transmission from one generation to the next with the highest genetic resolution possible, we developed a method to examine the mitotic divisions that occur in the *Drosophila* germline in order to determine the number of B chromosomes carried by a single adult (see STAR Methods). Since this assessment can be done after the individual is mated, it allowed us to directly measure the transmission of B chromosomes from a single parent to their progeny. We first applied our technique to measure the transmission frequency of B chromosomes that had been freshly introduced into our wild-type (WT) background (Figure S1A). Single, unmated females carrying B chromosomes were mated to males that did not carry B chromosomes, then assessed for B chromosome copy number using our ovary mitotic preparation protocol (Figures 1A and 1B; see STAR Methods). When all adult F1 progeny had emerged, we performed ovary mitotic preparations on several individual daughters from a single female, each of which is the result of a single meiotic event (Figure 1C).

The number of B chromosomes transmitted in each single meiotic event was converted into a percent by dividing the number of B chromosomes the F1 female carries by the number of B chromosomes her mother carries and multiplying it by 100. When we perform this analysis on multiple WT parental females and their progeny, the mean B chromosome transmission is consistent between individual females and is close to the expected 50% transmission if the B chromosomes are being inherited in a random (Mendelian) fashion (Figure 1D). We anticipated the B chromosomes would display Mendelian inheritance in this genetic background because the B chromosomes have been shown to not exhibit drive themselves, nor are they rapidly lost from a WT stock when there is no outcrossing.^19^

The transmission of B chromosomes from *mtrm*^*126*^*/TM3* females from the original stock was noticeably higher overall (Figure 1E). We combined the results from individual females within each genotype to obtain average B chromosome transmission frequencies of 52.15% and 63.77% for WT and *mtrm*^*126*^*/TM3*, respectively (Figure 1F). The mean B chromosome transmission frequency for WT is not significantly different from 50%, which is the expected mean in the absence of biased segregation (p = 0.1511, one sample t test, Data S1F), indicating the overall B chromosome transmission through WT females is Mendelian. Conversely, the B chromosome transmission frequency in the original stock is statistically significantly higher than through WT females (p < 0.0001, Welch’s unpaired t test), confirming that it is not the B chromosome that is promoting its own proliferation but rather the *mtrm*^*126*^*/TM3* genetic background that is permissive for B chromosome drive.

### Drive of the B chromosomes is strong in females but not in males

**Figure 2.**
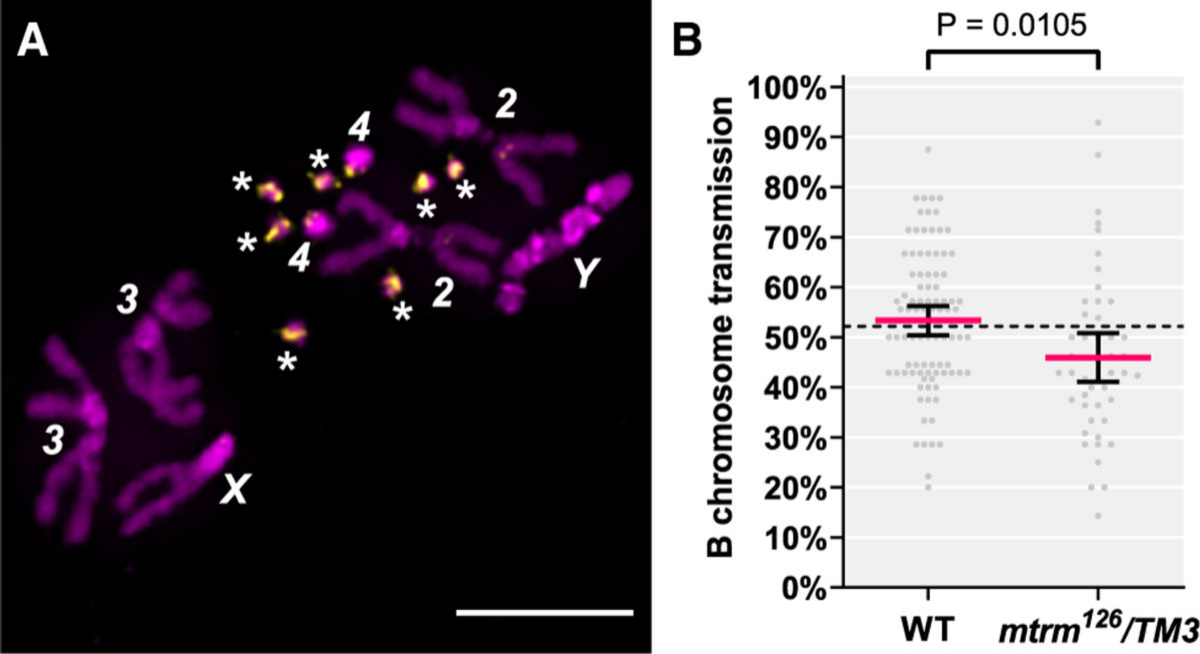
Males from the original *mtrm*^*126*^*/TM3* stock exhibit drag (A) Representative image of a metaphase from a testes mitotic preparation from a parental male with seven B chromosomes. Scale bar, 5 μm. (B) Cumulative transmission frequencies of the B chromosomes from *mtrm*^*126*^*/TM3* males from the original stock (n = 47) or wild-type (WT) (n = 91) males. The red line indicates the mean, error bars represent the 95% confidence interval of the mean. The p value is indicated in the figure (unpaired t test with Welch’s correction). The dotted line represents the B chromosome transmission frequency measured through WT females (52.15%). See also Figures S1 and S2 and Data S1A and S1G.

Though our analysis revealed that the drive of the B chromosomes is strong in females from the original stock, we were curious if males from the original stock were also able to transmit B chromosomes at an elevated frequency. To determine this, we applied our chromosome squash technique to the tips of the testes from males where the pre-meiotic mitotic divisions occur (Figure 2A; see STAR Methods). In a WT background, the B chromosome transmission frequency in males (53.32%) was almost identical to the transmission frequency observed in females (52.15%), reaffirming that the B chromosomes are unable to promote their own inheritance (Figure 2B).

Surprisingly, we observed that the B chromosomes in *mtrm*^*126*^*/TM3* males are subject to the opposite of drive, a phenomenon referred to as drag.^26^ Instead of being preferentially passed on, the B chromosomes are transmitted at a statistically significantly reduced frequency (45.92%) as compared with WT (53.32%) (Figure 2B; p = 0.01047, Welch’s unpaired t test), which was consistent across the parental males (Figure S2). This drag was initially surprising to us because *mtrm* is only expressed in the female germline and is not expressed anywhere in males.^3^ Given the complexities of male meiosis in *D. melanogaster* such as territory formation in prophase I, investigating how the B chromosomes segregate in spermatogenesis will be an interesting avenue of future research.^27^ Overall, since males exhibit drag of the B chromosomes, we conclude that the drive of the B chromosomes in the original stock is not general but is instead female-specific.

### Female drive of the B chromosomes is dependent on a genetic dose reduction of Mtrm in the presence of the *TM3* balancer chromosome

**Figure 3.**
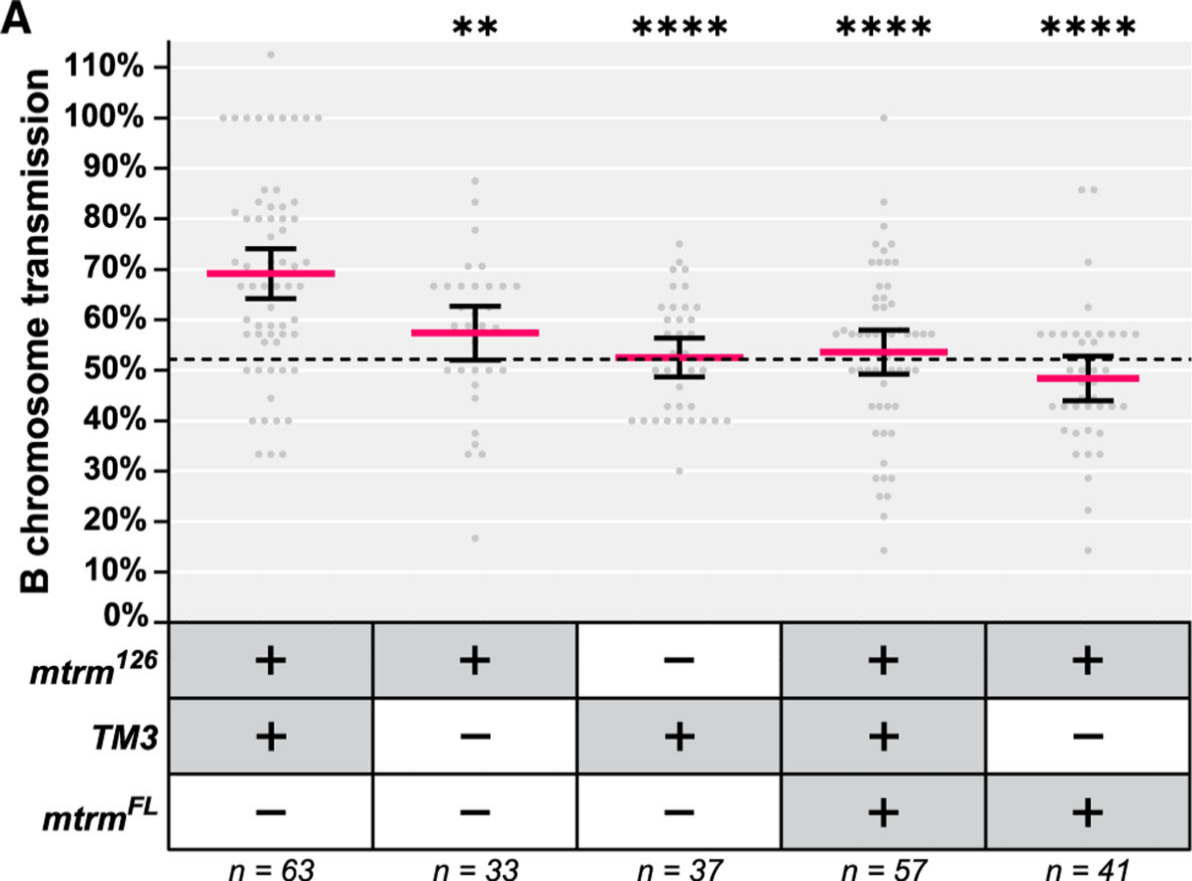
A genetic dose reduction in Mtrm is necessary but not sufficient for the strong drive of the B chromosomes (A) Cumulative transmission frequencies of the B chromosomes from females of the indicated genotypes. The red line indicates the mean, error bars represent the 95% confidence interval of the mean. After a comparison of all means via one-way ANOVA followed by Tukey’s multiple comparisons test, the mean B chromosome transmission frequency of recapitulated *mtrm*^*126*^*/TM3* females (first column) was significantly different from the other four genotypes; pairwise comparisons not indicated on the graph were found to be insignificant. *mtrm*^*FL*^ refers to a UAS-controlled transgene that expresses a full-length copy of *mtrm*. Asterisks indicate the p value of the comparison between the indicated genotype and the recapitulated *mtrm*^*126*^*/TM3* genotype (**p = 0.0072, ****p < 0.0001). The dotted line represents the B chromosome transmission frequency measured through wild-type (WT) females (52.15%). See also Figures S1, S3, and S4 and Data S1A and S1H.

Since Matrimony (Mtrm) is only present in the female germline and is critical for the proper segregation of non-crossover (achiasmate) chromosomes during meiosis,^3,23,24^ we suspected having only one functional copy of *mtrm* may be responsible for the biased transmission frequency of the B chromosomes we observed in the original stock. To test this, we outcrossed the original stock to a different stock carrying the *TM3* balancer but no *mtrm*^*126*^ or B chromosomes (Figure S1B). In the outcrossed *mtrm*^*126*^*/TM3* females, we observed robust B chromosome transmission that was at a slightly higher frequency than what we measured in the original *mtrm*^*126*^*/TM3* stock (69.17%, Figure 3). We suspect this modest increase is due to the removal of background suppressors of B chromosome drive after the outcross. When we measured B chromosome transmission frequency in *mtrm*^*126*^ females without *TM3*, it was statistically significantly reduced as compared with outcrossed *mtrm*^*126*^*/TM3* females (57.37%, Figure 3). Surprisingly, the B chromosome transmission frequency in *TM3* females without *mtrm*^*126*^ (52.52%) was also statistically significantly reduced as compared with the outcrossed *mtrm*^*126*^*/TM3* females and is almost identical to the transmission frequency we observed through WT females (Figure 3). Together, these results show that both the genetic dose reduction of Mtrm and the presence of the *TM3* balancer chromosome are necessary—but neither individually are sufficient—to produce permissive conditions for B chromosome drive.

To further investigate if *TM3*’s drive enhancement is from something intrinsic to *TM3* or from the presence of a balancer per se, we outcrossed the original stock to other stocks that carried different balancers and measured B chromosome transmission. We did not observe robust drive when *mtrm*^*126*^ was combined with other third chromosome balancers (*TM1* or *TM6B*), or a balancer for the second chromosome (*SM1*), indicating that this drive is specific to *TM3* and not dependent on the presence of a balancer per se (Figure S3). Additionally, the drive of the B chromosomes is the same regardless of whether the progeny inherited the *TM3* balancer or the *mtrm*^*126*^ chromosome, indicating that *TM3* inheritance is not influencing B chromosome inheritance (Data S1Q). These results lead us to hypothesize that *TM3*’s contribution to meiotic drive is genetic. It does not appear that *mtrm* expression from *TM3* is disrupted (Figure S4A), and there are no mutations within *TM3*’s copy of *mtrm* that change the amino acid sequence of the Mtrm protein. The *TM3* balancer could harbor a novel mutation in a critical component of the distributive system, have a disruption to a gene or its regulatory elements caused by any one of *TM3’*s inversion breakpoints,^28^ or have repurposed or restructured an unknown component that normally acts to promote the modest drive of the centromere on chromosome 3.^29^ Regardless, additional studies are necessary to determine the exact nature of *TM3’*s ability to synergize with *mtrm*^*126*^ and permit meiotic drive.

Though we are not aware of any other aberrations or genetic modifications on the chromosome carrying the *mtrm*^*126*^ null mutation, we wanted to confirm that the drive of the B chromosomes was specifically dependent on a genetic dose reduction of *Mtrm* and not from an unknown mutation that is linked to the *mtrm*^*126*^ chromosome. To do this, we expressed a functional copy of *Mtrm* from a transgenic construct under UASp-Gal4 control (referred to as *mtrm*^*FL*^) using the *nanos-GAL4* germline driver, a combination that has been demonstrated to replenish the genetic dose of Mtrm and rescue other phenotypic issues caused by the absence of Mtrm such as achiasmate chromosome nondisjunction and aberrant meiotic spindles.^24,30^ When the *mtrm*^*FL*^ transgene was expressed, drive of the B chromosomes was eliminated: 53.60% with the *TM3* balancer present, 48.38% when it was absent (Figure 3). This result confirms that a reduced genetic dose of Mtrm is necessary for B chromosome drive. Additionally, due to the critical role of Mtrm during female meiosis, we believe this drive is meiotic in nature and will herein refer to the biased transmission of the B chromosomes as female meiotic drive.

### The B chromosomes cluster abnormally in metaphase I-arrested oocytes, indicating a failure of the distributive segregation system

The female meiotic drive of the B chromosomes results in progeny inheriting more B chromosome copies than expected, but it remains unclear how these extra copies are initially deposited in the egg. During female meiosis, only one of the four meiotic products becomes the egg pronucleus and the other three are discarded in the rosette (polar body). This asymmetry is inherent to female meiosis and can be exploited by selfish genetic elements to bias their segregation toward the egg pronucleus and increase their overall transmission to progeny, much like the chromosome knobs in corn and enlarged centromeres in mice.^31,32^ When there is only one functional copy of *mtrm*, the distributive segregation system responsible for properly segregating non-crossover chromosomes (such as the B chromosomes) is impaired, which can result in abnormal chromosome arrangements during metaphase I and subsequent chromosome missegregation.^3,24^ Directly observing chromosome dynamics during the meiotic divisions would provide the most definitive evidence as to how the B chromosomes are being preferentially segregated, but these divisions are rapid and typically take place *in utero*, making them difficult to reliably observe.^33–38^

As an alternative approach to investigate B chromosome dynamics during meiosis, we cytologically examined the position of chromosomes at the metaphase I arrest in late-stage oocytes using fluorescent *in situ* hybridization (FISH). In prometaphase I, homologs that are held together with crossovers (chiasmata) become bioriented and localize to the center of the spindle. Achiasmate homologs, which are connected via heterochromatic threads instead of crossovers and rely on the distributive system for their segregation, are more dynamic and will traverse the spindle before becoming bioriented.^39,40^ As the homologs congress into a single DNA mass at the metaphase I arrest, their final orientation is highly correlated with how they will segregate in the first meiotic division.^41,42^ Therefore, we can observe the orientation of B chromosomes at this arrest in high-driving genotypes to determine if they are oriented properly (allocated evenly to either side of the DNA mass) or aberrantly (unevenly distributed or aligned improperly). The proper orientation of B chromosomes would suggest that their biased segregation occurs after release from the metaphase I arrest, such as when the spindle rotates to become perpendicular to the cortex and the meiotic divisions commence. If the B chromosomes exhibit an aberrant distribution or arrangement within the DNA mass at metaphase I arrest, then it would indicate a distributive system failure is contributing to the biased segregation of the B chromosomes.

**Figure 4.**
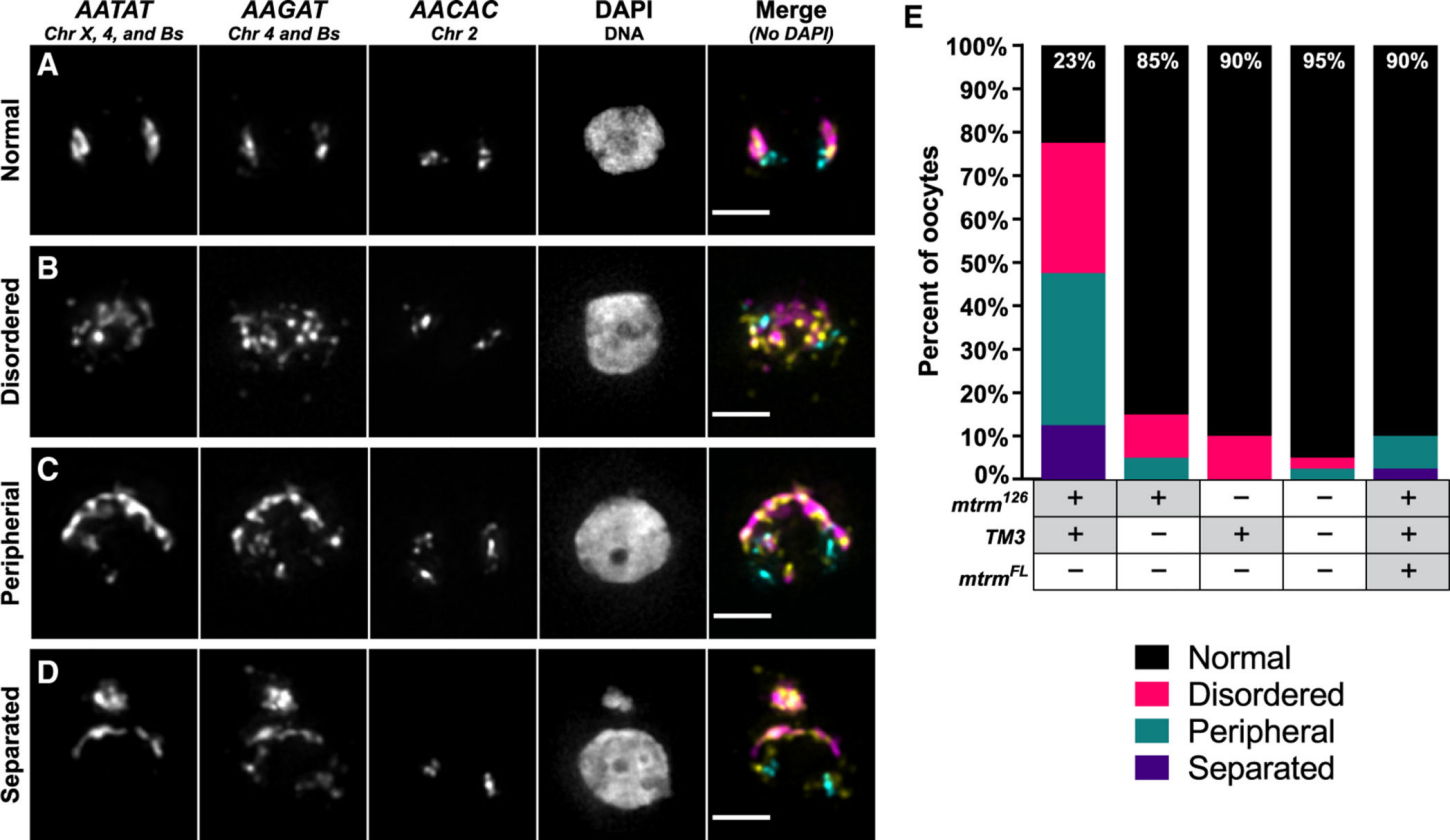
High meiotic drive of the B chromosomes correlates with increased abnormal B chromosome arrangements in metaphase I oocytes (A–D) Representative images of normal (A), disorganized (B), peripheral (C), or separated (D) B chromosome arrangements in oocytes arrested in metaphase I. Probes used for FISH recognize chromosomes X, 4, and the B chromosomes (*AATAT*, magenta in merged image), chromosome 4 and the B chromosomes (*AAGAT*, yellow in merged image), and chromosome 2 (*AACAC*, cyan in merged image). DNA (DAPI) is pictured but not included in the merged image. Scale bars, 2 μm. (E) Stacked bar chart indicating the proportion of oocytes observed for each chromosome arrangement. *mtrm*^*FL*^ refers to a UAS-controlled transgene that expresses a full-length copy of *mtrm*. For each sample, n = 40 except *mtrm*^*126*^ alone where n = 20. Values at the top of each bar indicate the percentage of oocytes that had normal chromosome arrangements. See also Figure S4 and Data S1I and S1J.

To readily identify the B chromosomes in the oocyte, we used FISH probes that recognize the *AATAT* satellite repeat (carried on the B chromosomes, chromosome X, and chromosome 4) and the *AAGAT* satellite repeat (carried on the B chromosomes and chromosome 4) (Figure S4B). As a control, we also used a FISH probe recognizing the *AACAC* satellite repeat that is located within the pericentromeric region of chromosome 2 to confirm the oocyte was in metaphase I and indicate the orientation of the meiotic spindle. We found that in the outcrossed *mtrm*^*126*^*/TM3* genetic background with strong meiotic drive, almost 80% of the oocytes we examined had an aberrant distribution of the B chromosomes. A normal distribution had the B chromosomes relegated to both ends of the DNA mass (Figure 4A), but abnormal distributions were categorized as disorganized, peripheral, or separated (Figures 4B–4D). B chromosomes that were scattered across the DNA mass were considered “disorganized,” whereas B chromosomes that were at the periphery of the oocyte were classified as “peripheral.” The last category, “separated,” indicates that a smaller mass of DNA that contained B chromosomes was physically separated from the DNA mass; this phenotype was enriched in oocytes collected from outcrossed *mtrm*^*126*^*/TM3* females, the genotype that shows the strongest meiotic drive. By contrast, abnormal B chromosome arrangements were found in a small percentage of oocytes (≤15%) from each of the *mtrm*^*126*^ alone, *TM3* alone, or WT backgrounds (Figure 4E). Consistent with this observation, the frequency of abnormal B chromosome arrangements is reduced to WT levels in the presence of a Mtrm transgene (Figure 4E). These results indicate that elements of the distributive system are failing to properly orient the B chromosomes leading up to the metaphase I arrest, potentially setting them up to be improperly segregated during the subsequent meiotic divisions.

The distributive segregation system has been long described as a “backup” mechanism to properly segregate, or distribute, chromosomes that failed to form crossovers.^21,43^ It is indispensable in *D. melanogaster* because chromosome 4 does not form crossovers, but evidence suggests that a distributive system is also operating in fission yeast and potentially in humans, neither of which carry constitutively achiasmate chromosomes.^44,45^ Hence, the distributive system may be more than just for backup, and the drive of the B chromosomes has led us to reexamine this paradigm and reconsider the purpose of this system.

Mutations in several genes involved in the distributive system, such as *nod* and *Axs*, result in high rates of nondisjunction of non-crossover chromosomes, but the distribution of these nondisjunction events is not random.^46,47^ This is also true for *mtrm* as the presence of only one functional copy leads to rampant (~30%) missegregation of chromosome 4 with a strong bias for meiotic products carrying two copies of chromosome 4 over zero copies at a frequency at (or exceeding) 2:1.^23^ When we transform chromosome 4 missegregation frequency into a measure of transmission frequency, we see that chromosome 4 transmission is Mendelian in a WT (49.99%) but is biased in *mtrm/*+ (54.80%). This trend is similar to what we observe for B chromosome transmission in WT (52.15%) and *mtrm*^*126*^ heterozygotes (57.37%), demonstrating the general ability of *Mtrm* to suppress drive and ensure unbiased chromosome segregation during meiosis. Though *mtrm* is poorly conserved on the sequence level outside of *Drosophila*, it has functional orthologs in yeast (Spo13 in budding yeast and Moa1 in fission yeast) and mammals (Meikin) that also interact with Polo kinase and promote proper chromosome segregation during meiosis.^30,48–51^ Taken together, we propose that *Mtrm* has an additional role as a meiotic drive suppressor and that the distributive system serves as a meiotic drive suppression apparatus that prevents invasive genetic elements from exploiting the inherent asymmetry of female meiosis.

## STAR★METHODS

### RESOURCE AVAILABILITY

#### Lead contact

Further information and requests for resources and reagents should be directed to and will be fulfilled by the lead contact, Stacey L. Hanlon (stacey.hanlon@uconn.edu).

#### Materials availability

All *Drosophila* stocks and reagents used in this study will be made available upon request without any restriction.

#### Data and code availability

- The data reported in this paper will be shared by the lead contact upon request. Original data underlying this manuscript can be accessed from the Stowers Original Data Repository at http://www.stowers.org/research/publications/libpb-1574
- This paper does not report original code.
- Any additional information required to reanalyze the data reported in this paper is available from the lead contact upon request.

### EXPERIMENTAL MODEL AND STUDY PARTICIPANT DETAILS

#### Fly stocks and husbandry

The complete genotype for all stocks used in this work can be found in Table S1. All test crosses were set using a mouth aspirator (no CO_2_) to avoid a delay in mating. Stocks and crosses were maintained on standard cornmeal media supplemented with active dry yeast and kept at 24°C and 70% humidity in constant light conditions. For transgenic experiments, we chose to induce expression of *mtrm* using the nanos promoter driving a 3x-FLAG-tagged full-length copy because it has been shown to best rescue chromosome nondisjunction and other defects resulting from a genetic dose reduction in *Mtrm*.^24,30,53^

For crosses measuring B chromosome transmission in females, virgin females were collected, aged for 3–5 days, and individually mated with a single non-B chromosome carrying wild-type male. After 5–7 days, the parental (P) males were discarded and the ovaries from individual P females were removed and squashed (see “squash preparation techniques” from adult ovaries below). After 18 days, we squashed ovary tissue from several F1 adult females; by this time, any adult F1 progeny will have eclosed but the F2 generation will not have reached adulthood. For measuring B chromosome transmission through males, males were collected, aged for 3–5 days, and individually mated with a single non-B chromosome carrying wild-type virgin female. After 24 hours, the testes from individual P males were squashed (see “squash preparation” from adult testes below), and the P female was allowed to remain in the vial to continue laying for another 4–6 days (5–7 days total) before being discarded.

### METHOD DETAILS

#### Squash preparation techniques

##### Ovary mitotic preparations

Ovary mitotic preparations (OMPs) were conducted as previously described.^20^ Mated adult females were anesthetized with CO_2_ until incapacitated (5–10 sec), then moved to a fresh 50 μL drop of 0.7% sodium chloride. Whole ovaries were removed and the carcass discarded. To enrich for pre-meiotic mitotic divisions, the tips of the ovaries were severed from later (stage 8 and beyond) stages. The ovary tips were hypotonically treated in 0.5% sodium citrate for five minutes, then fixed for 4 minutes in 2 mL of fixative solution (45% acetic acid, 2.5% paraformaldehyde) that was made fresh for each dissection session.

To squash the tissue, fixed ovary tips were moved to a 3 μL drop of 45% acetic acid on a siliconized coverslip and gently teased apart to spread out the tissue. A microscope slide was inverted onto the coverslip and pressed gently to spread the liquid to the edges of the coverslip. The slide and coverslip were squashed for 2 minutes using a hand clamp (48–22-3002; Milwaukee Tools), then directly placed into liquid nitrogen for at least 5 minutes. After a dissection session, slides were quickly removed from the liquid nitrogen one at a time and their coverslips were immediately popped off using a razor blade. The slide was then dehydrated by placing it in cold (−20 °C) 70% ethanol for at least 10 minutes, followed by cold (−20 °C) 100% ethanol for at least 10 minutes. Slides were removed and allowed to completely air dry, then stored in a corked slide box kept at room temperature.

##### Testes mitotic preparations

The procedure for testes mitotic preparations (TMPs) is similar to the OMPs above but with a few modifications. Individual males that had mated within the previous 24 hours were anesthetized with CO_2_ until incapacitated (5–10 sec), then moved to a fresh 50 μL drop of 0.7% sodium chloride. The entire reproductive tract minus the ejaculatory bulb was removed and the carcass was discarded. The remaining reproductive tract, which includes the testes, accessory glands, seminal vesicles, and ducts, was then hypotonically treated, fixed, and squashed as described for the OMPs above.

#### Fluorescent in situ hybridization (FISH)

Fluorescent *in situ* hybridization (FISH) was conducted as previously described.^20^ For each slide, 21 μL of a FISH solution (50% formamide, 10% dextran sulfate, 2x SSC, and 0.5 nM fluorophore-labeled probe) was applied directly to the dried tissue squash (see above). A glass coverslip was placed on top, and the slide was heated to 95 °C on a heat block for 5 minutes, followed by an overnight incubation (16–24 hr) at 30 °C in the dark. Slides were then washed three times for at least 15 minutes each in 0.1x SSC, blown dry with air from the house line, and mounted in 5 μL Vectashield with DAPI and covered with a coverslip that was sealed to the slide with nail polish.

#### Imaging parameters of tissue squashes

All images were acquired on an Applied Precision DeltaVision deconvolution microscope using a 100x objective with 1.6x auxiliary magnification. For each sample, stacks of 10 z-images with a thickness of 0.2 μm were taken using standard DAPI, FITC, TRITC, and Cy5 filter sets. Images were deconvolved using SoftWoRx v.6.1.3 or later (Applied Precision/GE Healthcare) following Applied Precision protocols.

#### Late-stage oocyte collection and FISH

Late-stage (stage 14) oocyte collection and treatment was performed according to published protocols.^54^ To enrich for oocytes arrested in metaphase I, 20 young (≤ 2 days old) females were collected and aged for four days without males in a vial with fresh yeast paste. Females were anesthetized with a brief exposure to CO_2_ then immediately decapitated to prevent hypoxia-induced changes to the oocyte.^55^ Whole ovaries were dissected from ~20 females in Modified Robb’s buffer (500 mM HEPES, 500 mM sucrose, 275 mM sodium acetate, 200 mM potassium acetate, 50 mM glucose, 6 mM magnesium chloride, 5 mM calcium chloride, pH adjusted to 7.4 with 11:8 sodium hydroxide:potassium hydroxide). Ovaries were allowed to settle, the buffer was removed, and ovaries were fixed in 550 μL fixation buffer (1x PBS, 150 mM sucrose) and 250 μL 16% formamide. Tubes were inverted manually for 2.5 minutes, then 800 μL of heptane was added and the tubes vortexed for 1 minutes. Ovaries were washed twice in 1 mL PBSTX-0.1% (1x PBS, 0.1% Triton X-100), then disrupted with a P1000 tip to separate the late-stage oocytes from the bulk of the ovary tissue. Free late-stage oocytes were allowed to settle, and the buffer with remaining tissue was removed and replaced with 500 μL PBSTX-0.1%. Oocytes were then washed for 5 minutes with 2x SSCT (2x SSC, 0.1% Tween-20).

To perform FISH, oocytes were transferred to a 500 μL tube, allowed to settle, and buffer was removed. The oocytes were stepped into formamide by adding 500 μL each of SSCT-20% (2x SSC, 20% formamide), then SSCT-40% (2x SSC, 40% formamide), and finally and SSCT-50% (2x SSC, 50% formamide), each time nutating for 10 minutes and allowing the oocytes to settle before removing the buffer and replacing it with the next in the series. After the third wash, the buffer was removed and replaced with fresh 500 μL SSCT-50%, then placed into a hybridization oven to rotisserie for four hours at 37 °C. After the incubation, the oocytes were allowed to settle, the buffer was removed and replaced with 40 L hybridization solution consisting of 18 μL formamide, 1 μL each probe, 1 μL water, and 18 μL salty DS (20% dextran sulfate, 4x SSC). Tubes were placed into thermalcycler set at 91 C with a deep-well head (to accommodate the 500 μL tubes) for 3 minutes, followed by a 37 C overnight incubation. The next day, the oocytes were rinsed then washed with 500 μL SSCT-50% pre-warmed to 37 °C and put into a hybridization oven to rotisserie for one hour in the dark at 37 °C. The oocytes were allowed to settle and the buffer was removed and replaced with 500 μL of SSCT-40% pre-warmed to 37 °C and placed back into the 37 °C hybridization oven to rotisserie for another hour in the dark. After the oocytes settled, the buffer was removed and replaced with 500 μL SSCT-20% and allowed to nutate for 10 minutes at room temperature in the dark. Oocytes were rinsed then washed in 500 μL 2x SSCT for 10 minutes at room temperature in the dark. DAPI was added to this wash with five minutes remaining to stain for DNA, followed by two more 10 minute washes in 2x SSCT. To mount, the oocytes were allowed to settle and most of the buffer was removed. The oocytes were transferred to a 22 mm x 22 mm No. 1.5 glass cover slip where residual buffer was carefully wicked away with a Kimwipe, then mounted in Prolong Gold (Invitrogen) by placing a 50 μL drop on a clean microscope slide and inverting it onto the coverslip. All slides were left to cure completely overnight at room temperature in the dark.

#### Imaging parameters of late-stage oocytes

All images were acquired on the Hawley Lab’s Applied Precision DeltaVision deconvolution microscope using a 100x objective with 1.6x auxiliary magnification. For each sample, stacks of 20 z-images with a thickness of 0.2 μm were taken using standard DAPI, FITC, TRITC, and Cy5 filter sets. Images were deconvolved using SoftWoRx v.6.1.3 or later (Applied Precision/GE Healthcare) following Applied Precision protocols.

Oocytes were determined to be at the metaphase I arrest point based on the following criteria: 1) dorsal appendages were mature in length; 2) nurse cells were completely reduced in size; 3) the DNA mass was one large, circular body comprising all the essential chromosome; 4) the *AACAC* FISH probe that hybridizes to the pericentromeric region of chromosome 2 had two discreet foci that were on opposite ends of the DNA mass, indicating the homologs were finished becoming bioriented. It is important to note that there is occasionally a second, smaller DNA mass that contained B chromosomes that was not at the poles of the mass; the presence of these deviant masses did not exclude the oocyte from being imaged.

#### Quantitative PCR analysis of *mtrm* expression

Virgin females from the original *mtrm*^*126*^*/TM3* stock were crossed to wild-type males to generate *mtrm*^*126*^/+ and +*/TM3* females. Females between 1–4 days old were collected from this cross, as well as from the wild-type and original *mtrm*^*126*^*/TM3* stock. Roughly 20 ovaries from each genotype were dissected in 1x PBS pH 7.4 and immediately placed in RNAlater solution on ice until all four samples were collected. RNA was extracted using Invitrogen’s PureLink RNA Mini Kit that included the optional on-column PureLink DNase step. Total RNA was quantified via NanoDrop, then cDNA was made from 1 mg of extracted RNA in a 20 μL reaction using the BioRad iScript cDNA Synthesis Kit that includes both random and oligo(dT) primers.

Each qPCR reaction was performed in triplicate using BioRad’s iTaq Universal SYBR Green Supermix with 2 μL of the appropriate first-strand cDNA synthesis reaction. Standard qPCR was performed on a BioRad CFX96 Real-Time PCR machine with the following cycle conditions: 95 °C for 3 minutes, 95 °C for five seconds, 60 °C for 30 seconds, read plate, repeat cycle 39 more times. The standard deviation of the reference target (*RpL32*^52^) between the four samples was less than 0.5. Primer efficiencies (105.9%, R2 = 0.999 for *RpL32*, 108.2%, R2 = 0.997 for *mtrm*) and dynamic range were determined under the same reaction and cycle conditions but using two-fold dilutions of genomic DNA. All analysis (Cq determination, expression analysis, etc.) was conducted using BioRad’s CFX Manager (Version 3.1.1517.0823).

### QUANTIFICATION AND STATISTICAL ANALYSIS

#### Statistical analysis

Statistical analyses were conducted using GraphPad Prism (version 9.4.1 for Windows, GraphPad Software, San Diego, California USA, www.graphpad.com). Comparisons between two data sets were performed using an unpaired t test with Welch’s correction, and multi-sample comparisons were analyzed with the appropriate ANOVA followed by unpaired t tests with the appropriate corrections for multiple comparisons.

#### B chromosome counting and transmission

The B chromosome copy number in each individual was assessed cytologically by examining mitotic metaphases in squashed germline tissue (see above). For each squash, we applied a FISH probe that recognizes the *AAGAT* satellite repeat enriched on the B chromosomes and chromosome 4 that allowed us to unequivocally determine B chromosome copy number in each metaphase.^20^ Since females have ~30 ovarioles per pair of ovaries, we were able to assess B chromosome copy number easily and consistently in ten metaphases for each OMP. Conversely, males only have two testes, making it very difficult to not only catch cells in pre-meiotic metaphase but also have enough scorable metaphases, therefore in each TMP we reduced our threshold to at least five pre-meiotic mitotic metaphases scored per TMP. The median of our counts for each squash served as the official B chromosome copy number for that individual. We then divided the number of B chromosomes carried by the F1 female by the number of B chromosomes her parent carried and multiplied it by 100, giving us the percent of B chromosomes transmitted.

All crosses set to measure B chromosome transmission had only one parent that carried B chromosomes to ensure all B chromosomes inherited by the progeny (F1) could only have come from the B-carrying parent. Therefore, the number of B chromosomes transmitted to each of the F1 progeny is the result of how the B chromosomes were distributed in a single meiosis within the B-carrying parent. We found no correlation between the number of B chromosomes a parent carried and the transmission of B chromosomes (Figure S4C). Unfortunately, we were only able to score adult female F1 progeny due to the infrequency of pre-meiotic mitotic metaphases from adult males (roughly 10–15% of TMPs had ≥ 5 scorable metaphases), though we do not have reason to suspect the B chromosome transmission differs based on sex of the progeny. Such a discrepancy would lead to sex-specific differences in B chromosome copy number within the stock population, which is not what we observe when we compare the adult male and female parents of the same genotype (Figure S4D). This result leads us to conclude that the chromosome content of the sperm does not impact the number of B chromosomes that are transmitted to the female pronucleus during the meiotic divisions.

At least five F1 OMPs were scored for each parent, and each parent must have had at least five B chromosomes. If a parent or F1 carried an abnormal number of essential chromosomes (for example, three copies of chromosome 4), the squash was not scored; this is to ensure that only normal meioses—those where there were the normal number of essential chromosomes in the parent and the correct number were passed to progeny—were part of the calculations. This restriction ensures consistent B chromosome transmission frequency determination across genotypes because the segregation of the essential chromosomes may not be independent of the B chromosomes as it is known that B chromosomes promote chromosome 4 nondisjunction.^19^ All values for the parental and F1 squashes, the B chromosome transmission calculations, and pertinent statistics can be found in Data S1A.

#### Late stage oocyte analysis

Stacks of deconvolved images were combined in a single z-projection showing maximum intensity, and images were selected at random for analysis. The orientation and arrest of each oocyte was verified by examining the location of the *AACAC* signals on each homolog of chromosome 2; if the *AACAC* signals were not on either side of the oocyte or if one focus appeared to be much brighter than the other, the oocyte was not scored. Next the *AAGAT* channel was examined and the arrangement of the B chromosomes was determined. Their position was categorized as either bilateral (the B chromosome signal was localized toward both presumptive poles), disordered (the B chromosomes are not in any defined arrangement and are scattered over and through the DNA mass), peripheral (the B chromosomes are positioned along the periphery of the DNA mass, forming a crescent shape), and separated (a discrete, separated DNA mass that is not at either pole contains the B chromosomes). All values for the number of scored oocytes can be found in Data S1I.

## Supplementary Material

MMC2

MMC1

## Figures and Tables

**Table:** KEY RESOURCES TABLE

REAGENT or RESOURCE	SOURCE	IDENTIFIER

Chemicals, Peptides, and Recombinant Proteins		

Sodium chloride	Sigma	Cat#S3014–1KG
Formamide	Sigma	Cat#47670
Sigmacote	Sigma	Cat#SL2–100mL
Vectashield + DAPI (1.5μg/mL)	Vector Laboratories	Cat#H-1200
Sodium citrate, dihydrate	Sigma	Cat#7810–1KG
Acetic acid (Glacial)	Sigma	Cat#A6283–1L
Paraformaldehyde, 16% aqueous	Electron Microscopy Sciences	Cat#15710
Dextran sulfate	Sigma	Cat#D8906–5G
20x Sodium chloride-sodium citrate (SSC)	Alfa Aesar (ThermoFisher)	Cat#AAJ60839K2
HEPES	Sigma	Cat#H4034–25G
Sucrose	Sigma	Cat#S0389–500G
Sodium acetate	Sigma	Cat#S2889–250G
Potassium acetate	Sigma	Cat#P1190–100G
Glucose	Sigma	Cat#G7021–100G
Magnesium choloride	Sigma	Cat#M8266–100G
Sodium hydroxide	Sigma	Cat#S5881–500G
Potassium hydroxide	Sigma	Cat#221473–500G
10x Phosphate buffered saline (PBS)	Sigma	Cat#6505–4L
Triton X-100	Sigma	Cat#T8787–250ML
Tween 20	Sigma	Cat#P9416–100ML
Prolong Gold	Invitrogen	Cat#P36934
RNAlater	Invitrogen	Cat#AM7021
PureLink DNase	Invitrogen	Cat#12185010

Critical Commercial Assays		

PureLink RNA Mini Kit	Invitrogen	Cat#12183018A
iScript cDNA Synthesis Kit	BioRad	Cat#1708891
iTaq Universal SYBR Green Supermix	BioRad	Cat#1725121

Experimental Models: Organisms/Strains		

*Drosophila* stocks used in crosses to measure B chromosome transmission, see Table S1	This manuscript	N/A

Oligonucleotides		

[AlexaFluor 647]-*AATATAATATAATATAATATAATATAATAT*	Integrated DNA Technologies	N/A
[AlexaFluor 547]-*AAGATAAGATAAGATAAGATAAGATAAGAT*	Integrated DNA Technologies	N/A
[AlexaFluor 488]-*AACACAACACAACACAACACAACACAACAC*	Integrated DNA Technologies	N/A
*TTCACGATCTTGGGCCTGTATG* (Forward primer for *RpL32* (*rp49*))	Eurofins Genomics (sequence from Chang et al.^52^)	N/A
*TTGTTGTGTCCTTCCAGCTTCA* (Reverse primer for *RpL32* (*rp49*))	Eurofins Genomics (sequence from Chang et al.^52^)	N/A
*CAACGAAGGTGCATCCCAAG* (Forward primer for *mtrm*)	Eurofins Genomics	N/A
*GAGTCATCCGAACAGGTATCCG* (Reverse primer for *mtrm*)	Eurofins Genomics	N/A

Software and Algorithms		

SoftWoRx v 6.1.3 or later	Applied Precision/GE Healthcare	https://download.cytivalifesciences.com/cellanalysis/download_data/softWoRx/6.5.2/SoftWoRx.htm
Prism v 9.4.1	GraphPad	www.graphpad.com
FIJI	ImageJ2	https://imagej.net/software/fiji/
CFX Manager Version 3.1.1517.0823	BioRad	https://www.bio-rad.com/en-us/sku/1845000cfx-manager-software?ID=1845000&WT.mc_id=220128033438&WT.srch=1&WT.knsh_id=4b41044f-64f1-4426-8c40-8451a07ba515&gclid=Cj0KCQjwuLShBhC_ARIsAFod4fIFpXqwPMyHZG72eGSQXHknoESlccoyjHWMZnEARYZq4ko2mCqJ7NcaAkqgEALw_wcB

Other		

Hand clamp for squashing tissue	Milwaukee Tools	48–22-3002

## Highlights

- Female meiotic drive promotes biased B chromosome inheritance in *D. melanogaster*
- A genetic reduction of *Mtrm* and the *TM3* balancer chromosome are necessary for drive
- Strong drive correlates with aberrant B chromosome arrangements during metaphase I
- The distributive segregation system may act to suppress drive during female meiosis
