# Supplementary material for: B chromosomes reveal a female meiotic drive suppression system in Drosophila melanogaster
Source: Curr Biol. Author manuscript; Available in PMC 2023 Jun 8. (PMC10247447; doi:10.1016/j.cub.2023.04.028)
Supplement: MMC1 [file NIHMS1895322-supplement-MMC1.pdf]

Current Biology, Volume *33*

Supplemental Information

B chromosomes reveal a female meiotic drive suppression system in *Drosophila melanogaster*

Stacey L. Hanlon and R. Scott Hawley

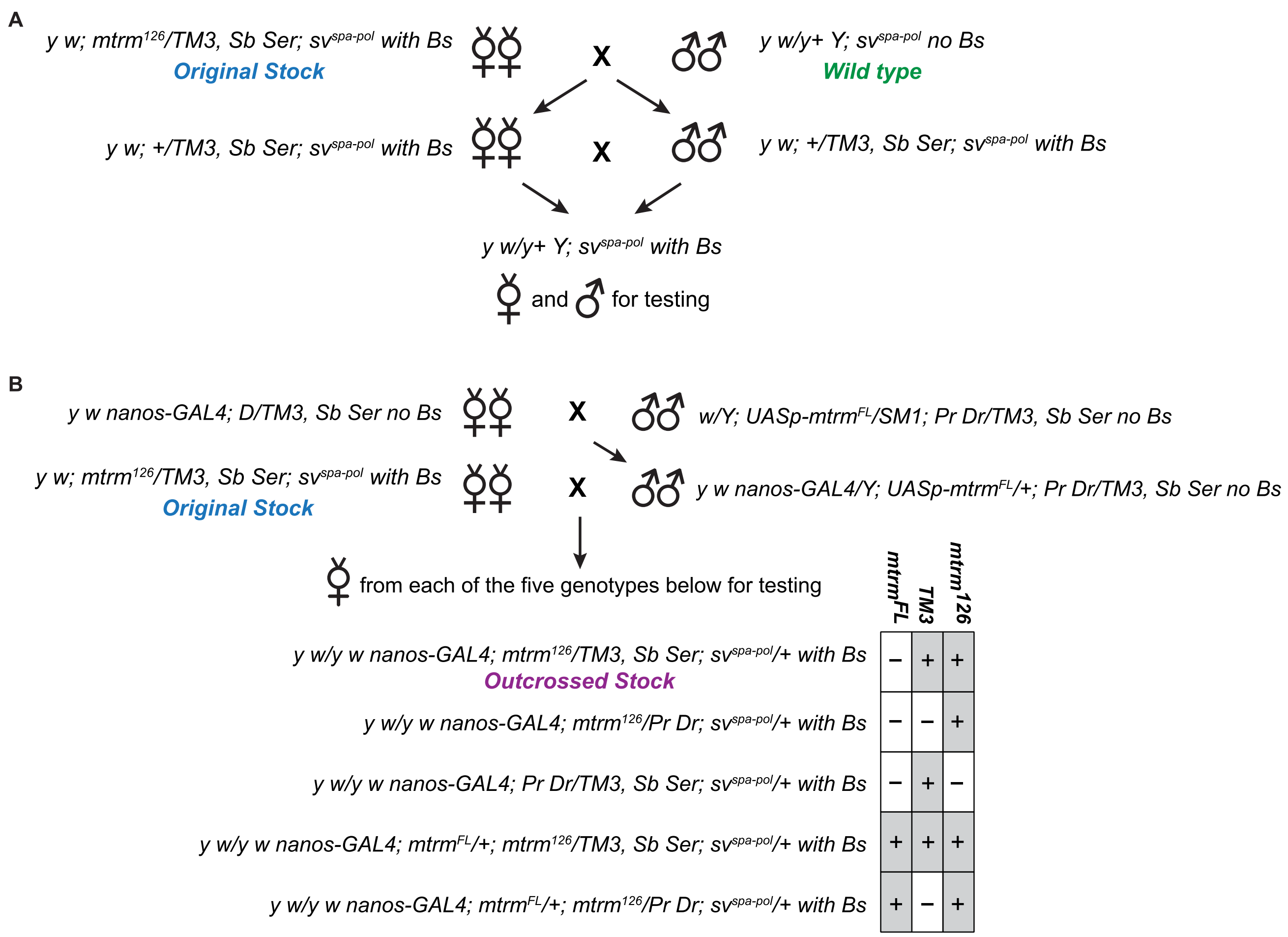

Figure S1. Cross schemes to introduce B chromosomes into various genetic backgrounds. Related to Figures 1-4.

A) Cross scheme used to introduce B chromosomes into a wild-type (WT) genetic background. This background carries three well-characterized recessive markers: *yellow* (*y*), *white* (*w*), and *shaven* ($sv^{spa\text{-}pol}$). B chromosomes were always introduced through $mtrm^{126}/TM3$ females. B) Cross scheme used to obtain the genotypes tested in Figures 3 and 4. Homologous chromosomes are separated by a forward slash, and homologous chromosome sets are separated by semicolons. Female symbol has a "v" added to indicate females used for the cross were unmated (virgins). Homozygous homologs are listed once (without a forward slash). All known mutations and phenotypic markers are listed, and all genes not listed are considered to be wild type. A complete list of genotypes is available in Table S1.

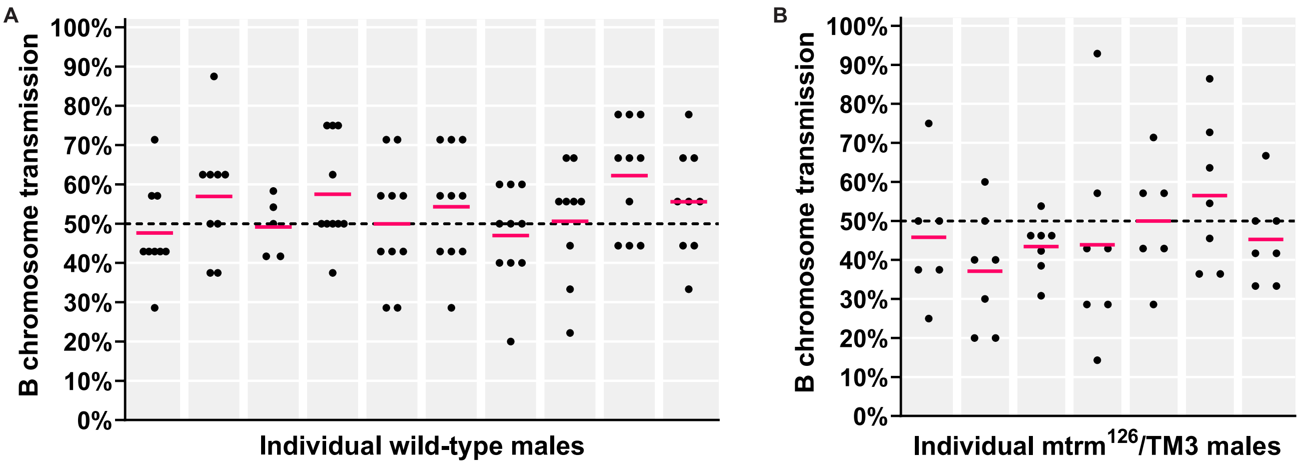

Figure S2. Individual male parents. Related to Figure 2.

A) Transmission frequencies of B chromosomes between wild-type parental males and their progeny, plotted by individual male. Red line indicates the mean transmission frequency for that individual parental male. Dotted line is set at 50%, which is the expected transmission frequency if B chromosome segregation was Mendelian. B) Same as in (A) but parental males are from the original B chromosome stock and are *mtrm*^{126}/*TM3*. Within each genotype, there was no significant difference between the means of the parental males (Welch's ANOVA produced a non-significant P value of 0.3509 for wild-type and 0.6021 for *mtrm*^{126}/*TM3* males). See also Data S1K and S1L.

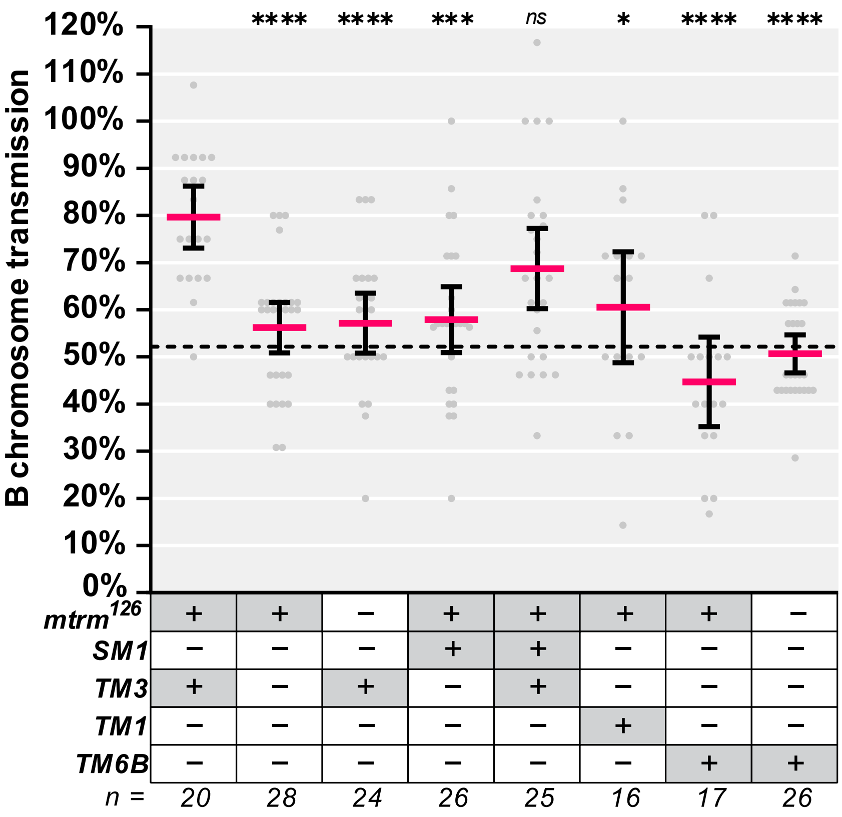

Figure S3. Drive of the B chromosomes is strongest in a *mtrm*^{126}/*TM3* genetic background. Related to Figure 3.

The original B chromosome stock was outcrossed to stocks balanced for either Chromosomes 2 and 3 or Chromosome 3 alone to create the genotypes shown above. Sample sizes are shown below the table. Asterisks indicate the P value of the comparison between the indicated genotype and the recapitulated *mtrm*^{126}/*TM3* genotype (*ns* = not significant, * = 0.0405, *** = 0.0002, **** < 0.0001; Welch's ANOVA followed by Dunnett's T3 multiple comparisons test). Dotted line represents the B chromosome transmission frequency measured through wild-type females (52.15%). See also Table S1 for a full listing of stocks and genotypes and Data S1M.

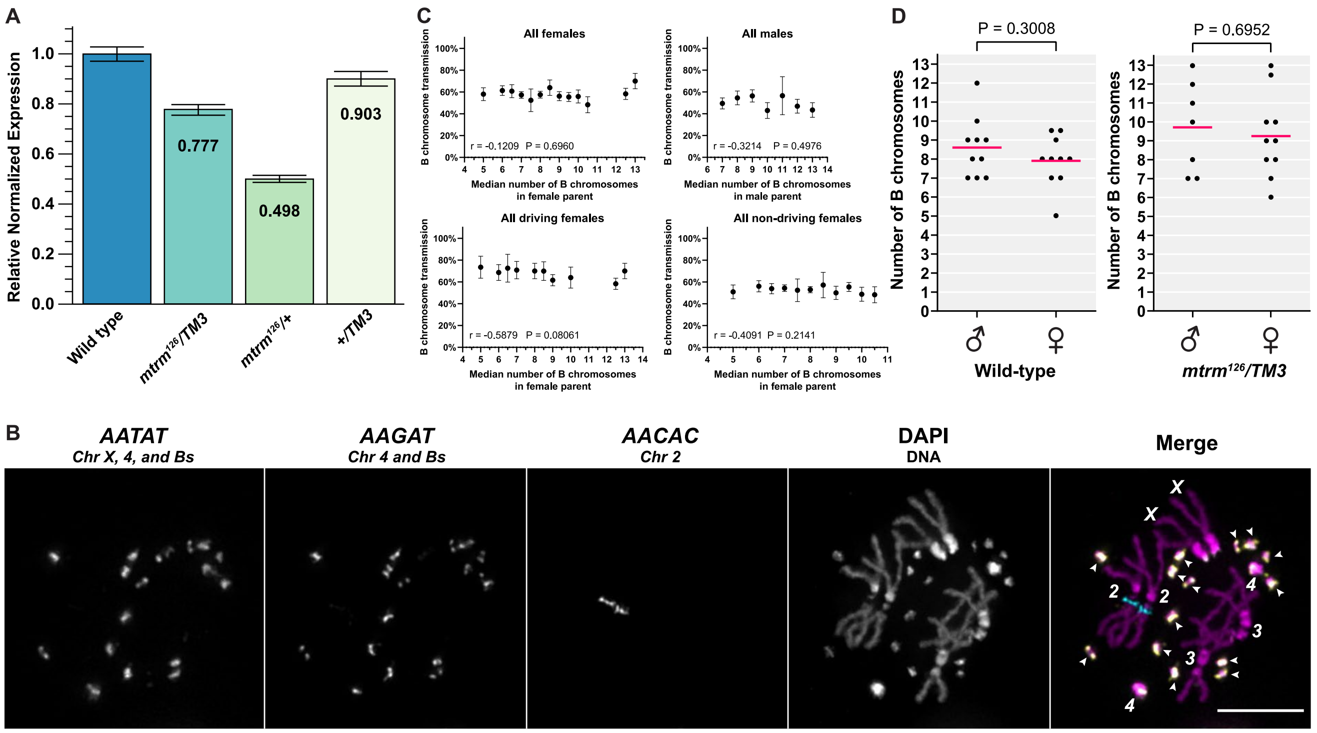

Figure S4. Supporting information for *mtrm* expression, FISH probe cytogenetics, and B chromosome transmission dynamics. Related to Figure 3, Figure 4, and STAR Methods.

A) Quantitative PCR (qPCR) analysis of *mtrm* expression in ovaries from 1-4 day-old females of the indicated genotypes. Values displayed are the normalized *mtrm* expression relative to the control (wild type) sample, which itself is normalized to 1. B) Probes used for FISH in metaphase I oocytes were hybridized to pre-meiotic mitotic chromosomes obtained from ovary mitotic preparations to verify the location of their target on each chromosome. In our genetic background, the probes recognize Chromosomes X, 4, and the B chromosomes (*AATAT*, gray in merged image), Chromosomes 4 and the Bs (*AAGAT*, yellow in merged image), and Chromosomes 2 (*AACAC*, cyan in merged image). DNA is in magenta (DAPI). B chromosomes are denoted with arrowheads. Bar = 5 µm. C) The median number of B chromosomes in the parent is not correlated with the transmission frequency of B chromosomes to progeny. Female parents are broken down further based on whether they exhibit drive. Spearman r coefficient and P values are indicated in each graph. The driving

females show a weak negative correlation that is not statistically significant; this trend may be due to drive being stronger after an outcross. D) The parental B chromosome copy number is consistent between males and females of the same genotype. Comparison of B chromosome copy number in wild-type and original *mtrm*^{126}*/TM3* stock parental males and females used in this study. Red line indicates the mean number of B chromosomes. Sample size is n = 10 except for *mtrm*^{126}*/TM3* males where n = 7. No significant difference was identified between the sexes (P values indicated in figure; unpaired t test with Welch's correction). See also Data S1N-P.

Stock name (as listed in Data S1A)	Established stock or created from cross?	DSLH No. (for stocks)	Carries Bs?	Genotype
$mtrm^{126}$/TM3 (original stock)	Stock	17	YES	*y w/y+Y; $mtrm^{126}$/TM3, Sb Ser; $sv^{spa-pol}$*
WT (wild type)	Stock	5	NO	*y w/y+Y; $sv^{spa-pol}$*
nos-GAL4	Stock	10	NO	*P{GAL4::VP16-nanos.UTR}, y w/B^{S}Y; D/TM3, Sb Ser*
$mtrm^{FL}$	Stock	16	NO	*w; P{$y^{+t7.7}$ w^{+mC}=UAS-3xFLAG-MtrmDmel}attP40/SM1; Pr Dr/TM3, Sb Ser*
Double-balanced stock	Stock	13	NO	*w; Sp/SM1; Pr Dr/TM3, Sb Ser*
nos;$mtrm^{126}$/TM3 (outcrossed stock)	From cross (see Figure S1)	N/A	YES	*P{GAL4::VP16-nanos.UTR}, y w/y w; $mtrm^{126}$/TM3, Sb Ser; $sv^{spa-pol}$/+*
nos;$mtrm^{126}$/Pr	From cross (see Figure S1)	N/A	YES	*P{GAL4::VP16-nanos.UTR}, y w/y w; $mtrm^{126}$/Pr Dr; $sv^{spa-pol}$/+*
nos;Pr/TM3	From cross (see Figure S1)	N/A	YES	*P{GAL4::VP16-nanos.UTR}, y w/y w; Pr Dr/TM3, Sb Ser; $sv^{spa-pol}$/+*
nos;$mtrm^{FL}$;$mtrm^{126}$/TM3	From cross (see Figure S1)	N/A	YES	*P{GAL4::VP16-nanos.UTR}, y w/y w; P{$y^{+t7.7}$ w^{+mC}=UAS-3xFLAG-MtrmDmel}attP40/+; $mtrm^{126}$/TM3, Sb Ser; $sv^{spa-pol}$/+*
nos;$mtrm^{FL}$;$mtrm^{126}$/Pr	From cross (see Figure S1)	N/A	YES	*P{GAL4::VP16-nanos.UTR}, y w/y w; P{$y^{+t7.7}$ w^{+mC}=UAS-3xFLAG-MtrmDmel}attP40/+; $mtrm^{126}$/Pr Dr; $sv^{spa-pol}$/+*
y w/w;Sp/+;$mtrm^{126}$/Pr	From cross	N/A	YES	*y w/w; Sp/+; $mtrm^{126}$/Pr Dr; $sv^{spa-pol}$/+*
y w/w;Sp/+;$mtrm^{126}$/TM3	From cross	N/A	YES	*y w/w; Sp/+; $mtrm^{126}$/TM3, Sb Ser; $sv^{spa-pol}$/+*
y w/w;Sp/+;Pr/TM3	From cross	N/A	YES	*y w/w; Sp/+; Pr Dr/TM3, Sb Ser; $sv^{spa-pol}$/+*
yw/w;+/SM1;$mtrm^{126}$/Pr	From cross	N/A	YES	*y w/w; +/SM1; $mtrm^{126}$/Pr Dr, Sb Ser; $sv^{spa-pol}$/+*
yw/w;+/SM1;$mtrm^{126}$/TM3	From cross	N/A	YES	*y w/w; +/SM1; $mtrm^{126}$/TM3, Sb Ser; $sv^{spa-pol}$/+*
TM1/TM6	Stock	BDSC 1794	NO	*TM1/TM6B, Tb[1]*
$mtrm^{126}$/TM1	From cross	N/A	YES	*y w/Y; $mtrm^{126}$/TM1; $sv^{spa-pol}$/+*
$mtrm^{126}$/TM6B	From cross	N/A	YES	*y w/Y; $mtrm^{126}$/TM6B, Tb[1]; $sv^{spa-pol}$/+*
TM3/TM6B	From cross	N/A	YES	*y w/Y; TM3, Sb Ser/TM6B, Tb[1]; $sv^{spa-pol}$/+*

Table S1. Stock list and genotypes tested in this work. Related to the STAR Methods.
